# The Clinical Significance of Serum Free Light Chains in Bladder Cancer

**DOI:** 10.3390/jcm12093294

**Published:** 2023-05-05

**Authors:** Monika Gudowska-Sawczuk, Jacek Kudelski, Michał Olkowicz, Grzegorz Młynarczyk, Piotr Chłosta, Barbara Mroczko

**Affiliations:** 1 Department of Biochemical Diagnostics, Medical University of Bialystok, Waszyngtona 15A St., 15-269 Bialystok, Poland; 2 Department of Urology, Medical University of Bialystok, M. Skłodowskiej-Curie 24A St., 15-276 Bialystok, Poland; 3 Department of Urology, Jagiellonian University Medical College, Jakubowskiego 2 St., 30-688 Kraków, Poland; 4 Department of Urology, Medical University of Vienna, Währinger Gürtel 18-20 St., 1090 Vienna, Austria; 5 Department of Neurodegeneration Diagnostics, Medical University of Bialystok, Waszyngtona 15A St., 15-269 Bialystok, Poland

**Keywords:** free light chains, FLC, kappa, lambda, cancer, bladder cancer, biomarker

## Abstract

This research aimed to assess the clinical usefulness of serum kappa (κ) and lambda (λ) free light chains (FLCs) in patients with bladder cancer (BC). One hundred samples were collected and analysed from healthy volunteers (C) and bladder cancer patients. Cancer patients were divided into two subgroups: low-grade (LG) and high-grade cancer (HG). Concentrations of FLCs, CEA, CA19-9, creatinine and urea were measured per manufacturers’ guidelines. The concentrations of κ and λ FLCs and CEA were significantly higher in BC patients in comparison to the control group. Moreover, the concentrations of κ and λ FLCs and CEA were significantly higher in both low-grade as well as high-grade cancer in comparison to the controls. The levels of κ and λ FLCs differed between tumour grades, with patients presenting higher concentrations in high-grade compared to low-grade cancer. In the total study group, κFLC correlated with λFLC, the κ:λ ratio, CRP, CEA, CA19-9, creatinine and urea. There was also a correlation between λFLC and κFLC, CRP, CEA, creatinine and urea. The λFLC showed a higher ability (sensitivity and PPV) to detect bladder cancer in comparison to κFLC and CEA. In addition, λFLC had a higher ability to exclude BC (specificity and NPV) than κFLC and CEA. λFLC also showed the highest accuracy in the detection of bladder cancer. In conclusion, the revealed differences in the concentrations of both κ and λ FLCs suggest their potential participation in bladder cancer development. Increased concentrations of free light chains in bladder cancer patients and the association with the tumour grade suggest that κ and λ FLC measurements may be useful in the diagnosis and prognosis of bladder cancer. This is the first research that evaluates the concentration of FLCs in bladder cancer, so further studies are necessary to confirm their usefulness as tumour markers of this malignancy.

## 1. Introduction

Bladder cancer (BC) is one of the most common urogenital cancers with a high mortality rate. The bladder cancer development risk is almost four times higher among men than women [1]. The list of risk factors includes, for example, smoking, type 2 diabetes, the exposure of the bladder area to ionizing radiation and the chronic inflammation of the bladder. Moreover, the chance of bladder cancer increases with age, and nearly 70% of diagnosed patients are people aged 65 and higher [2,3,4]. The most common BC symptoms are the appearance of painless, but massive, hematuria; the urgent need to pass urine; and pollakiuria.

Currently, the diagnosis of bladder cancer includes ultrasound examination, cystoscopy and the invasive puncturing of suspicious sites for histopathological examination. Urine cytology is recommended as a supplement to cystoscopy, but the diagnostic specificity of urine cytology is high only in high-grade bladder cancer. On the other hand, a negative result does not exclude the presence of cancer. Unfortunately, the possibilities of laboratory diagnostics concerning bladder cancer are limited. Thus, finding a quick and easy non-invasive biomarker seems to be very important because the timing of the diagnosis is crucial in further treatment, prognosis and patient survival [5,6,7].

Local inflammation, which may spread throughout the body, is associated with the presence of factors that trigger the immune system’s response. In addition to infections or tissue damage, tumours may also cause inflammation [8]. In the course of cancer and other inflammatory conditions, increased immunoglobulin synthesis is observed very often. Additionally, the production of antibodies is always accompanied by a slight excess synthesis of the kappa (κ) and lambda (λ) immunoglobulin light chains, which are not bound to the heavy chain. Small amounts of free light chains (FLCs) are released into peripheral and may also be found in various body fluids such as urine. They are quickly filtered by the glomeruli andare normally present in the urine only in trace amounts. Physiologically, approximately 500 mg per day of FLCs are produced with a κ:λ ratio equal to approximately 2:1. At the time of the excessive production of FLCs, the reabsorption capacity of the renal tubules may be exceeded, resulting in the accumulation of FLCs in the serum [9,10]. This can occur in many clinical conditions, including chronic inflammation, immunological disorders, kidney failure and cancer. Increased levels of FLCs have been observed in different body fluids of patients with, e.g., multiple sclerosis and breast, lung or gastric cancers [11,12,13,14,15]. However, it should be noted that, as far as we are aware, serum FLCs have never been evaluated in the samples of patients with bladder cancer. Therefore, this study aimed to assess the significance and diagnostic utility of free light chains in bladder cancer.

## 2. Material and Methods

### 2.1. Study Design

We performed a research study involving patients with bladder cancer and healthy volunteers. Samples were collected between May 2022 and December of the same year. Each participant in the study had their kappa and lambda free light chain concentrations measured. At the same time, we determined the concentrations of CEA and CA19-9 as comparative markers. Then, we compared the results of the tested parameters between healthy controls and patients with bladder cancer and between different stages of cancer.

### 2.2. Subjects

Patients with bladder cancer were admitted to the Department of Urology of the Medical University of Bialystok. Subsequently, 60 patients who were referred for further transurethral resection of bladder tumour (TURBT) were enrolled in this study. Patients included in the study had a bladder tumour detected by cystoscopy and ultrasound. Fourteen patients had TURBT in the past. No other active cancer diseases were found. Tumour staging and classification were conducted per European Association of Urology guidelines. Patients with bladder cancer were divided into two subgroups: low-grade (*n* = 52%) and high-grade (*n* = 48%). In addition, according to the depth of tumour invasion (T) three groups of patients were distinguished: Ta (52%), T1 (15%) and T2 (23%).

Tested patients were males (*n* = 45) and females (*n* = 15) (age range: 56–80, mean age: 66.8).

The diagnosis of bladder cancer was based on the patient’s symptoms, ultrasound examination, cystoscopy and the result of the histopathological examination.

### 2.3. Control Group

Overall, 40 healthy volunteers (males *n* = 19, females *n* = 21) were admitted to our institution as the control group (age range: 23–75, mean age: 48.3). For each potential participant in the control group, the following exclusion criteria were applied: other comorbidities that can affect free light chain concentrations; pathological changes in the urinary system; active infections, both viral and bacterial.

Informed consent was obtained from all patients with bladder cancer and healthy volunteers. The study was approved on 25 June 2020 by the Bioethical Committee at the Medical University of Bialystok (APK.002.240.220).

### 2.4. Blood Sampling

Blood specimens were taken by vein puncture. Serum samples were obtained by centrifugation. Then, serum samples were aliquoted and frozen at −80 °C until they were analysed.

### 2.5. Methods

The concentrations of serum kappa and lambda-free light chains were measured on the Optilite analyser (The Binding Site, Birmingham, UK) with the use of the turbidimetric method. Determinations were performed under the manufacturer’s guidelines.

Detection ranges of serum FLCs listed in instructions:

κFLC—2.90–127.00 mg/L;

λFLC—5.20–139.00 mg/L;

κ:λ ratio—0.26–1.65.

Concentrations of CEA, CA19-9, CRP, creatinine and urea were measured on the Alinity analyser (Abbott) following the manufacturer’s recommendations. CEA and CA19-9 were assessed using the chemiluminescent microparticle immunoassay (CMIA) method. CRP was measured using the immunoturbidimetric method, and creatinine and urea were measured with the use of the enzymatic method. Detection ranges for the afore-mentioned parameters were 1.73–1500.00 ng/mL, 2.06–1200.00 U/mL, 1.00–480.00 mg/dL, 2.5–400.00 mg/dL and 3.00–125.00 mg/dL. In cases of concentrations below the lower limit of detection, the lowest values of the detection were used.

### 2.6. Statistical Analysis

Statistical data analysis has been performed using the Statistica 13.3 analytics software. The Mann–Whitney U test was used for the evaluation of the differences between the tested groups. The Spearman rank correlation test was used to check the association between tested variables. Cut-off values for κFLC, λFLC and CEA were calculated by Youden’s index as a criterion for selecting the optimum cut-off points.

*p* values < 0.05 were considered statistically significant.

## 3. Results

### 3.1. Patients’ Ages

The ages of the control group’s participants were significantly lower than the ages of the patients with bladder cancer (*p* < 0.005).

### 3.2. Serum Concentrations of Free Light Chains and Tumour Markers

**Figure 1:**
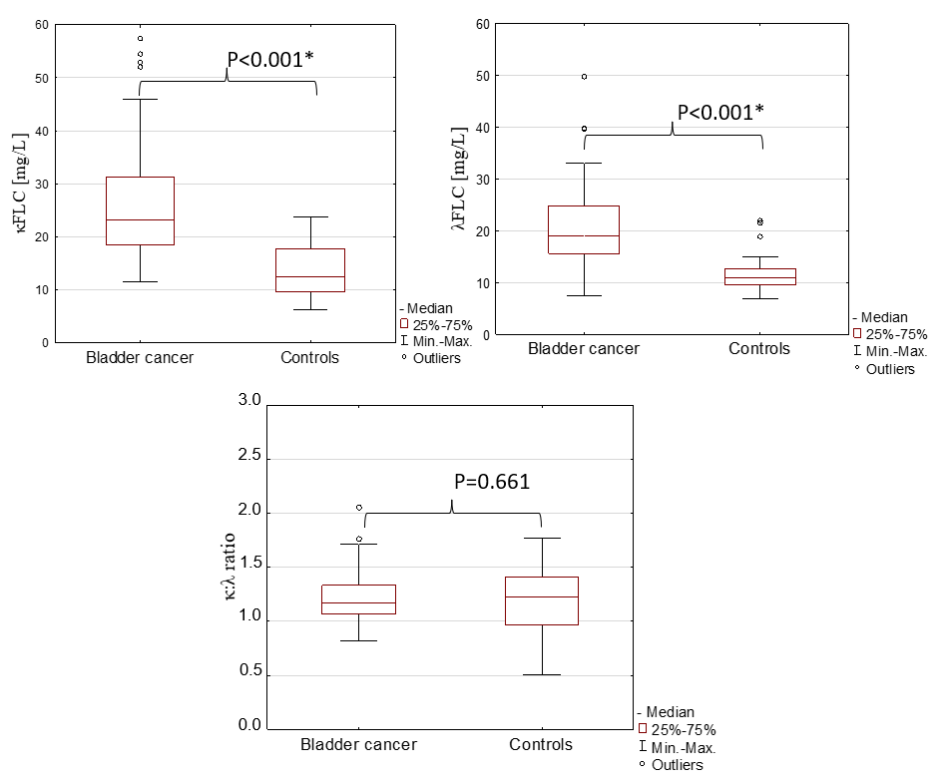
Serum concentrations of FLCs and the κ:λ ratio in patients with bladder cancer and controls. *—the significant differences between tested groups.

The results of serum κ and λ FLCs, the κ:λ ratio, CEA and CA19-9 in the patients with bladder cancer and the healthy controls are presented in Table 1. Figure 1 is the visual presentation of the serum concentrations of FLCs as well as the κ:λ ratio.

The serum concentrations of κFLC and λFLC differed significantly between the tested groups (*p* < 0.001 for both). Similarly to the κFLC and λFLC concentrations, the level of CEA was significantly higher in bladder cancer patients in comparison to the healthy controls (*p* < 0.001). The values of the κ:λ ratio and CA19-9 were similar in the bladder cancer patients and the controls (*p* = 0.661 and *p* = 0.206, respectively).

**Figure 2:**
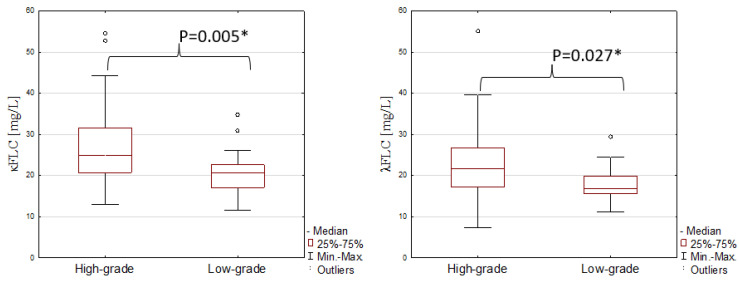
Serum concentrations of κFLC and λFLC in high- and low-grade bladder cancer. *—the significant differences between tested groups.

The statistical analysis revealed that the median serum concentrations of κFLC and λFLC depend on the tumour grade (Figure 2).

The medians of κFLC and λFLC concentrations were higher in high-grade bladder cancer (25.16 and 21.79 mg/L, respectively) than in low-grade (20.55 and 16.73 mg/L; *p* = 0.005 and *p* = 0.027, respectively). The median value of the κ:λ ratio and the concentrations of CEA and CA19-9 did not differ between the low (1.16, 2.87 ng/mL and 5.69 U/mL, respectively) and high grades of cancer (1.19, 2.27 ng/mL and 6.37 U/mL, respectively).

Moreover, the concentrations of κFLC, λFLC and CEA were significantly lower in the control group in comparison to low- (*p* < 0.001 for all comparisons) and high-grade cancer (*p* = 0.017, *p* < 0.001 and *p* < 0.001, respectively). The concentrations of CA19-9 and the κ:λ ratio did not differ between the control group and low-grade bladder cancer (*p* = 0.953 and *p* = 0.732, respectively) or high-grade bladder cancer (*p* = 0.183 and *p* = 0.676, respectively).

The serum concentrations of the free light chains and CEA according to the tumour infiltration depth (T) are presented in Table 2. The median of κFLC was significantly lower in Ta in comparison to T1 (*p* = 0.033) and T2 (*p* = 0.050). The λFLC concentration differs significantly between Ta and T1 (*p* = 0.005), but there was not any difference between Ta and T2 (*p* = 0.357). The concentrations of κFLC and λFLC were similar between T1 and T2 (*p* = 0.703 and *p* = 0.657, respectively). There was no difference in CEA concentration between Ta and T1 (*p* = 0.475), between Ta and T2 (*p* = 0.310) and between T1 and T2 (*p* = 0.182).

### 3.3. Correlations of Free Light Chains with Other Tested Parameters

Correlations between κFLC, λFLC, κ:λ ratio, CRP, CEA, CA19-9, creatinine and urea are presented in Table 3. Sprearman’s rank correlation test demonstrated that κFLC correlated with all tested parameters whereas λFLC did not correlate with κ:λ ratio and CA 19-9. κ:λ ratio correlated only with κFLC concentration.

### 3.4. Diagnostic Power of κ and λ Free Light Chains

The diagnostic usefulness of κFLC, λFLC and CEA in bladder cancer is presented in Table 4. The λFLC and κFLC showed a higher ability to detect bladder cancer (a sensitivity of ~80.00% for both) in comparison to CEA. The λFLC had slightly higher PPV than κFLC and CEA (90.30 vs. 82.80 and 82.50, respectively). λFLC also showed the highest ability to exclude bladder cancer, with an 83.80% specificity and an 88.60% negative predictive value.

**Figure 3:**
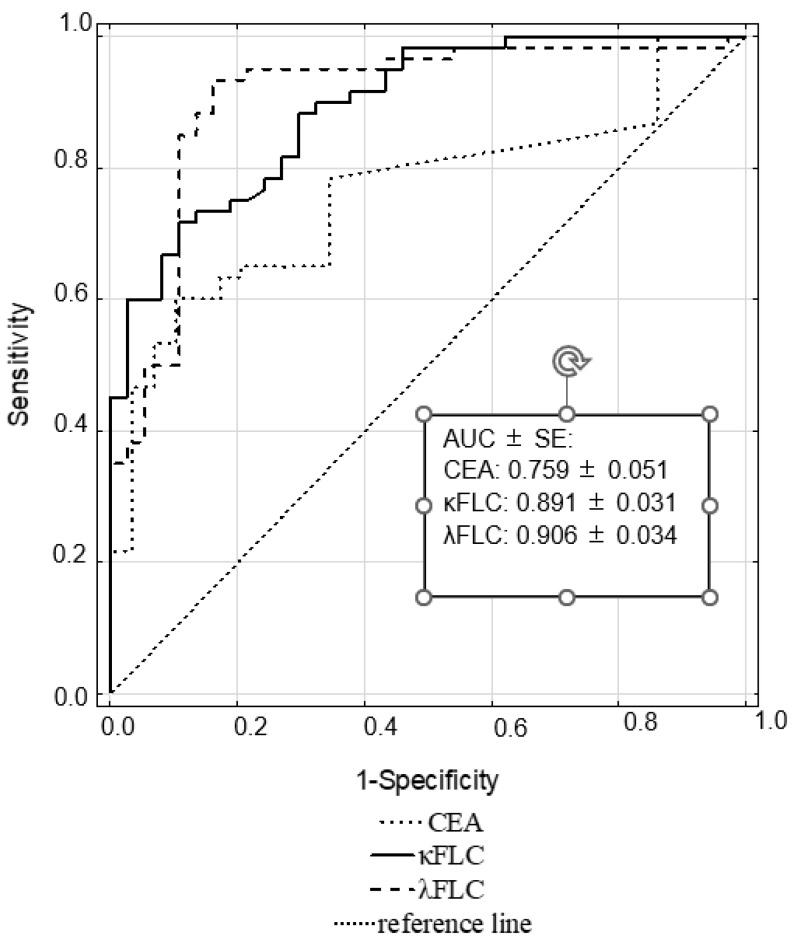
ROC curves for CEA, κ and λ free light chains in bladder cancer.

The ROC curve analysis indicated that the λFLC showed excellent discrimination ability (AUC = 0.906), whereas the test quality of κFLC and CEA in the detection of bladder cancer was good (Figure 3).

## 4. Discussion

When imaging tests (e.g., ultrasound through the abdominal wall) do not show an unequivocal picture of a neoplastic lesion in the bladder, cystoscopy is recommended. It is the most popular diagnostic test for a suspected bladder tumour. The procedure consists of inserting a cystoscope with a vision system through the urethra, which enables the visual assessment of the walls of the bladder and the collection of material for histopathological examination. However, it is an invasive test, and finding non-invasive laboratory markers of bladder cancer is necessary [5,6,16]. The most frequent laboratory test used, both in the diagnosis and in the follow-up of the patient after treatment, is urine sediment cytology. It consists of the microscopic evaluation of the urine sediment obtained from the patient and the detection of exfoliated cancer cells released from the tumour into the lumen of the bladder, which are then excreted in the urine. In recent years, some tumour markers in the urine have also been examined. It has been suggested that, e.g., the nuclear matrix protein 22 (NMP22) BladderChek test may be used for the detection of bladder cancer; however, its sensitivity, specificity and diagnostic usefulness have not been unequivocally confirmed [17,18]. Moreover, the role of blood tumour markers in bladder carcinoma is still not well established. However, carcinoma embryonic antigen (CEA) and carbohydrate antigen 19-9 (CA19-9), for example, have been evaluated in bladder cancer, and it has been observed that their levels correlate with tumour stage and grade.

Despite the significant development of diagnostic methods, there is still a steady increase in the incidence and mortality rates of malignant neoplasms in the world. Therefore, new markers allowing the earlier diagnosis of bladder cancer, more accurate assessments of the disease stage and the better monitoring of therapy is still being sought.

It is known that chronic inflammation underlies many diseases, including malignancies. During immune system activation, in the process of antibody synthesis, free light chains are produced by B lymphocytes. The clinical and diagnostic significance of free light chain measurements in the course of monoclonal gammopathies is currently the best-known and most thoroughly researched method that has been confirmed by numerous studies [19,20,21]. Additionally, increased levels of FLCs have been observed in the course of, e.g., breast, lung and gastric cancers [12,13,14,15]. However, free light chain concentrations, according to our best knowledge, were never evaluated in bladder carcinoma. Thus, all things considered, we decided to evaluate the level of free light chains in the serum of bladder cancer patients.

We have shown that the concentrations of κ and λ FLCs were almost twice as much in the serum of bladder cancer patients as the healthy subjects. On the other hand, the κ:λ ratio did not differ between tested groups, which may suggest the occurrence of polyclonal immunoglobulin FLC synthesis during bladder cancer caused by chronic inflammation. In addition, we observed higher levels of κ and λ FLCs in high-grade bladder cancer, so it seems that the concentration of FLCs is associated with tumour grade and disease progression. This may be related to the effect of FLCs on the cells of the immune system. On the other hand, the concentrations of κFLC and λFLC were similar between T1 and T2. However, this may be a result of the unequal distribution of patients in the groups, depending on the depth of tumour invasion (T).

It has been observed that many cells of the immune system, including mast cells, enter the tumour microenvironment, and previous studies have shown that mastocytes might be activated by free light chains alone. Mast cells are multifunctional cells that are part of the innate immune system, and they can have anti- and pro-tumorigenic effects. The pro-tumorigenic effects of mast cells include, e.g., their participation in the stimulation of angiogenesis and the degradation of the extracellular matrix, which facilitates the migration of cancer cells and immunosuppression reactions via the secretion of inflammatory factors. Thus, it seems that bladder cancer development may be indirectly related to the level of FLCs, which are over-produced during inflammation and may excessively activate mast cells. Evidence of this may be the presence of free light chains found by other researchers in the areas of mast cell infiltration. On the other hand, the inhibition of FLCs and, as a result, mast cells appears to be a promising therapeutic target for the treatment of cancers [22,23,24,25,26].

Moreover, we observed that FLC concentrations correlate with CRP and CEA levels. Knowing that CRP is a strong reactive acute phase protein and that CEA may be increased in cases of chronic inflammation and cancer, we can conclude that free light chains are closely related to inflammatory responses during bladder cancer progression [27,28]. In addition, the positive correlation between FLCs and elevated levels of creatinine and urea may reflect the obstruction of the flow in the urinary tract caused by cancer.

Among all tested parameters, serum λ and κ free light chain levels had the highest diagnostic value for bladder cancer. Hence, easy-to-perform quantitative measurements of free immunoglobulin light chains may be important blood markers of bladder cancer. We, therefore, carefully suggest that the determination of free light chain concentrations may improve diagnoses and may be used for the differentiation of low- and high-grade bladder cancers.

## 5. Limitations of the Study

Due to the exclusion criteria applied, the participants in the control group were younger than the patients with bladder cancer. Older patients yield a higher risk of different cancers that may have an impact on, e.g., the CEA concentrations. In addition, some patients also had TURBT in the past, and this should be stated as a limitation and as a possible bias in our study. Moreover, the synthesis of kappa and lambda chains occurs in various inflammatory disorders, so FLC measurements should be taken into consideration not only as a single test but also as a marker of bladder cancer, ultrasound examination and urine cytology. There is a lack of prior research on this topic, so it is hard to understand the role of FLCs in the pathomechanism of bladder cancer development. Because there is no other study that evaluates the usefulness of FLCs in bladder cancer, further studies are needed to confirm their diagnostic and clinical significance.

## 6. Conclusions

This is the first study that evaluated the significance of serum free light chains in bladder cancer. We showed that the concentrations of free light chains are significantly elevated in bladder cancer and that the levels of κ and λ FLCs increase proportionally with the grade of bladder cancer. In addition, a positive correlation between FLCs with CRP and CEA may reflect the immune system response during cancer development. In conclusion, it seems that serum κ and λ FLCs measurements may be helpful in the diagnosis of bladder cancer with very high diagnostic accuracy.

## Figures and Tables

**Table 1:** The results of serum κ and λ FLCs, the κ:λ ratio, CEA and CA19-9 in patients with bladder cancer and healthy controls.

	Variable Tested	κFLC [mg/L]	λFLC [mg/L]	κ:λ Ratio	CEA [ng/mL]	CA19-9 [U/mL]
Bladder cancer (A)	Median (min–max values).	23.09 ^B^* (11.50–137.00)	18.98 ^B^* (7.36–55.10)	1.17 (0.82–3.26)	2.60 ^B^* (1.72–18.83)	7.34 (2.05–256.43)
Control group (B)		12.36 ^A^* (6.28–23.70)	10.86 ^A^* (6.84–22.00)	1.22 (0.51–1.77)	1.73 ^A^* (0.66–4.60)	4.18 (2.06–19.38)

^A^, Bladder cancer; ^B^, control group; *—the significant differences between tested groups.

**Table 2:** Serum concentrations of free light chains and CEA according to the tumour infiltration depth (T).

		κFLC [mg/L]	λFLC [mg/L]	CEA [ng/mL]
Ta (A)	Median (min–max values).	19.42 ^B,C^* (11.50–52.06)	16.68 ^B^* (10.28–33.09)	2.56 ^B^* (1.73–12.16)
T1 (B)		23.16 ^A^* (15.05–86.27)	21.21 ^A^* (12.99–39.80)	3.39 ^A^* (1.73–18.83)
T2 (C)		29.86 ^A^* (14.47–137.00)	21.71 (7.36–28.44)	1.96 ^A^* (1.73–3.42)

^A^, Ta; ^B^, T1; ^C^, T2. *—the significant differences between tested groups.

**Table 3:** Spearman correlations between tested variables in the total study group.

Total Study Group	κFLC	λFLC	κ:λ Ratio	CRP	CEA	CA19-9	Creatinine	Urea
κFLC								
r		0.843	0.311	0.458	0.289	0.319	0.353	0.450
p		<0.001 *	<0.001 *	<0.001 *	0.004 *	0.002 *	0.001 *	<0.001 *
λFLC								
r	0.843		−0.121	0.397	0.272	0.199	0.385	0.350
p	<0.001 *		0.213	<0.001 *	0.007 *	0.052	<0.001 *	0.001 *
κ:λ ratio								
r	0.311	−0.121		0.120	0.047	0.178	−0.003	0.197
p	<0.001 *	0.213		0.272	0.650	0.084	0.977	0.080
CRP								
r	0.458	0.397	0.120		0.226	0.140	0.139	0.165
p	<0.001 *	<0.001 *	0.272		0.035 *	0.198	0.231	0.157
CEA								
r	0.289	0.272	0.047	0.226		0.262	−0.156	−0.111
p	0.004 *	0.007 *	0.650	0.035 *		0.009 *	0.146	0.320
CA19-9								
r	0.319	0.199	0.178	0.140	0.262		0.168	0.212
p	0.002 *	0.052	0.084	0.198	0.009 *		0.120	0.058
Creatinine								
r	0.353	0.385	−0.003	0.139	−0.156	0.168		0.514
p	0.001 *	<0.001 *	0.977	0.231	0.146	0.120		<0.001 *
Urea								
r	0.450	0.350	0.197	0.165	−0.111	0.212	0.514	
p	<0.001 *	0.001 *	0.080	0.157	0.320	0.058	<0.001 *	

Correlation ratio (r): 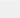
—0.000–0.100; 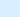
—0.101–0.300; 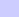
—0.301–0.500; 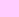
—0.501–0.700; 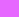
—0.701–0.900. *—significant correlation between tested variables.

**Table 4:** The diagnostic significance of serum κ and λ FLCs in bladder cancer.

	Cut-Off from the ROC	Sensitivity [%]	Specificity [%]	PPV [%]	NPV [%]	ACC [%]
κFLC [mg/L]	16.43	88.30	70.30	82.80	78.80	81.40
λFLC [mg/L]	13.38	93.30	83.80	90.30	88.60	89.70
CEA [ng/mL]	1.74	78.30	65.50	82.50	59.40	74.20

PPV, positive predictive value; NPV, negative predictive value; ACC, accuracy.

## Data Availability

The data that support the findings will be available on request under the corresponding author’s e-mail: monika.gudowska-sawczuk@umb.edu.pl.
